# Disorder-aided pulse stabilization in dissipative synthetic photonic lattices

**DOI:** 10.1038/s41598-019-49259-x

**Published:** 2019-09-09

**Authors:** Stanislav Derevyanko

**Affiliations:** School of Electrical and Computer Engineering, Ben Gurion University of the Negev, Beer Sheva, 84105 Israel

**Keywords:** Electronics, photonics and device physics, Optical physics

## Abstract

We consider a discrete time evolution of light in dissipative and disordered photonic lattice presenting a generalization of two popular non-Hermitian models in mathematical literature: Hatano-Nelson and random clock model and suggest a possible experimental implementation using coupled fiber loops. We show that if the model is treated as non-unitary Floquet operator rather than the effective Hamiltonian the combination of controlled photon loss and static phase disorder leads to pulse stabilization in the ring topology. We have also studied the topological invariant associated with the system and found additional evidence for the absence of Anderson transition.

## Introduction

Non-Hermitian systems with non-unitary time evolution have always attracted particular attention due to a large number of unusual dynamical effects observed there^1^. Non-hermitian systems are now commonplace at such diverse areas of physics as vortex dynamics in superconductors^2^, Dicke superadiance^3,4^ biological networks^5^ and photonic waveguides^6–9^ just to name a few.

The non-Hermiticity in quantum mechanics is usually associated with loss mechanism of some description or coupling to an unspecified reservoir with large number of degrees of freedom (heat bath). However recently a renewed interest to non-Hermiticity has been aroused due to the concept of PT-symmetric systems where the Hamiltonian albeit being non-Hermitian admits purely real spectrum of eigenvalues thus making the propagation conservative even if the individual eigenmodes are not orthogonal^10^. Arguably the field of application where the non-Hermitian effects are easiest to engineer and observe is that of optics^11–13^. Two important classes of optical systems suggested for these purposes are photonic meshes^14^ and coupled fiber loops^15^ collectively known as synthetic photonic lattices (SPLs)^16^. Both setups have their advantages and drawbacks but both provide the necessary ingredients for non-trivial non-conservative propagation effects predicted for non-Hermitian systems: (i) phase modulation acting as phase-randomized coupling and (ii) adjustable balance of gain and losses (providing non-Hermicity and in some cases PT symmetry see^17^). The phase modulation can be introduced in controllable pseudorandom way such that if it changes at each spatial position and each discrete time step it provides a minimalistic model for the effects of decoherence^15,18^ while when the induced pseudorandom phase modulation is static (i.e. does not change with the discrete time) the system experiences Anderson localization (AL)^18–22^. While the phenomenon of AL and the effect of disorder on pulse propagation are very well studied and understood in Hermitian systems (see e.g.^23^) the results in the non-Hermitian settings are still comparatively scarce (a few exceptions are provided by cases studied in^2,5,24^). The main reason for this is of course the variety of various ways in which the Hermicity can be broken. While recently an interesting connection between the Anderson transition and a topological charge in a large class of non-Hermitian systems has been discussed in^25^ the whole subject of localization in the non-Hermitian systems remains an open research topic.

In this paper we study the interplay of gain/dissipation and disorder on the stroboscopic evolution of pulses in synthetic photonic lattices. We suggest a possible implementation of two known non-Hermitian models: Hatano-Nelson (HN) model^2^ that is purely dissipative and a Feinberg-Zee random clock (RC) model^26,27^ which describes complex nearest neighbour coupling with randomized phases. Since we are interested here in the interplay of disorder and losses we introduce a combined model (which we call a generalized HN model, GHN) and propose a possible experimental setup where such a model can be realized. The setup is largely inspired by recent experimental studies of disorder and decoherence in the so-caled discrete time quantum walks (DTQW)^15,18,28^ and as such presents just one possible option. Note however that in our work the fiber loop configuration is used not in order to emulate the two components of a pseudo-spinor describing a random walker but rather to introduce the gain/loss imbalance and random phase mismatch between the retarded and advanced components of the same pulse. In these settings we show that strong phase disorder can act to approximately stabilize the pulse propagation by making the eigenvalues of the step-evolution (Floquet) operator localize around a unit circle even if it is generally non-unitary. The effect appears to be sensitive to the boundary conditions of the SPL as are many properties of disordered lattices (see e.g. recent paper^29^). While the eigenvalues can be tightly localized by disorder a large proportion of eigenmodes remain delocalized which is attested by the lack of the topological transition (see^25^). We also study how the presence of disorder affects the steering of the pulse introduced by asymmetric coupling in the HN model and describe the effects of disorder on the spreading of the initially localized pulse.

Note that other photonic realizations of HN model have been reported in literature – see the recent refs^30–32^. but as an effective non-Hermitian Hamiltonian ${ {\mathcal H} }_{eff}$ with the diagonal disorder or pseudoperiodic potential in the spirit of the original HN suggestion^2^ and the emphasis was on the localization properties of the pulse dynamics. In our implementation the model is realized as a *evolutionary step operator* i.e. $\exp (\,-\,i{ {\mathcal H} }_{eff})$. In these settings it has a different physical meaning and we show that the robustness of the pulse propagation is achieved because of (and not despite of) the unitary phase disorder. In addition we show here that in the model with random complex coupling there is no topological transition when the system experiences finite gain/loss.

## Results

### Theoretical model

Let us begin with stating the main evolution model studied in this paper. In the next section we discuss a possible photonic implementation with coupled fiber loops but at present let us introduce it in the general context. Consider a discrete evolution of a complex amplitude ${a}_{n}^{m}$ which can play the role of an amplitude of a single quantum particle in a dissipative system or a complex amplitude of a classical short light pulse in a photonic lattice. It is assumed that the the particle/pulse is observed stroboscopically at discrete time intervals labeled by *m* (which can be viewed as counting the number of round trips in a loop or a cavity) at a discrete set of time or coordinate positions labeled by the second index *n*. The dynamics of our model is specified by the following map:

1 $${a}_{n}^{m+1}=\frac{i}{2}({e}^{i{\varphi }_{\uparrow }(n)}\,{e}^{\gamma }\,{a}_{n+1}^{m}+{e}^{i{\varphi }_{\downarrow }(n)}\,{e}^{-\gamma }\,{a}_{n-1}^{m})$$

where *γ* > 0 represents anti-symmetric nearest neighbour gain/loss while the random phases *ϕ*_↑↓_(*n*) are assumed to be i.i.d. drawn from a uniform distribution on the interval [−Φ_*max*_, Φ_*max*_]. Small values of Φ_*max*_ correspond to small levels of disorder while the maximum value Φ_*max*_ = *π* corresponds to simultaneous random rotation of the complex coupling coefficients and will referred to as “strong disorder”.

As we shall see below the spectrum of the step-evolution operator and hence the dynamical properties of the system are sensitive to the boundary conditions. Having in mind the suggested fiber loop implementation of the synthetic photonic lattice it is natural to use periodic boundary conditions where *a*_*n*±*N*_ = *a*_*n*_ where *N* is the size (period) of the system. In the synthetic mesh picture of e.g.^14^ this corresponds to rolling the mesh into cylinder geometry. As for the phases *ϕ*_↑↓_, following refs^19,20^ we choose the model of static phase disorder studied earlier in the context of lossless (unitary) SPLs.

The model (1) has a connection with popular non-Hermitian spectral models considered in literature. To see this we can look for quasi-stationary solution of the evolution equation in the form ${a}_{n}^{m}={(iz)}^{m}\,{u}_{n}$ where *z* is a complex number. Substituting this ansatz into (1) we find that *u*_*n*_ is a solution of the following eigenvalue problem:

2 $$z{u}_{n}=\frac{1}{2}[{e}^{i{\varphi }_{\uparrow }(n)}\,{e}^{\gamma }\,{u}_{n+1}+{e}^{i{\varphi }_{\downarrow }(n)}\,{e}^{-\gamma }\,{u}_{n-1}]$$

with *u*_0_ = *u*_*N*_, *u*_*N*+1_ = *u*_1_ and the matrix in the r.h.s. defines the evolutionary step operator $\hat{U}$. Without phase modulation Φ_*max*_ = 0 this matrix is identical to that of Hatano-Nelson (HN) model^2^ originally suggested for describing vortex pinning in superconductors. In the opposite limit of passive network *γ* = 0 and Φ_*max*_ = *π* one obtains a “random clock” (RC) model first suggested in ref.^26^ and later extensively studied numerically in^27^. The general case presented by Eqs (1) and (2) combines the features of both models and we shall call it here a generalized Hatano-Nelson model (GHN) (or the Hatano-Nelson model with random complex coupling). Note that similar generalization of the HN model with off-diagonal *real* disorder was recently suggested and studied in^5^ in the context of random neural connection in biological networks.

It is important to emphasize that the physical meaning of the operator $\hat{U}$ considered here is that of the unit time step and not the effective Hamiltonian as it was understood in the previous condensed matter, photonic or biological applications of the HN model (see e.g.^2,5,8,31–33^). Therefore rather than discussing the non-Hermicity of the underlying effective Hamiltonian we shall look into the *non-unitarity* of the evolution operator $\hat{U}$ and its implications on the pulse dynamics. Both RC and HN operators regardless of the choice of the boundary conditions are not just non-Hermitian but non-unitary: their eigenvalues generally are not concentrated on the unit circle |*z*| = 1 leading to amplification or extinction of the individual modes. However as we shall demonstrate here in the combined GHN the periodic boundary conditions can rearrange the spectrum and make it quasi-unitary despite the original system lacking a distinct PT or chiral symmetry^14,29^.

## Suggested Photonic Implementation

**Figure 1:**
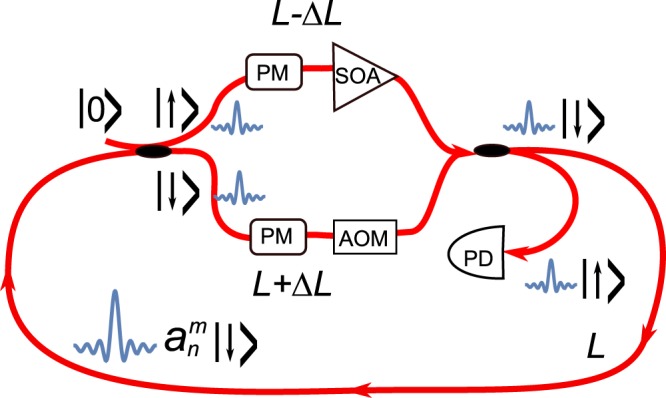
A schematic view of a suggested fiber setup.

In order to implement the GHN model (as well as its special cases) as an evolutionary step model we suggest a photonic setup shown in Fig. 1 and largely inspired by the ones considered in refs^15^ and^17^. A short pulse is launched into a single mode fiber span of length *L*. Both ends of the span are coupled to the two arms with the widths *L* − Δ*L* and *L* + Δ*L* respectively each containing a phase modulator (PM) and an active element which is either a semiconductor optical amplifier (SOA) or acusto-optical modulator (AOM). The fiber coupler introduces an even power split and a *π*/2 phase shift between signals in the two arms. Similarly to the setup of ref.^15^ the second port of the second splitter is coupled to a photodetector thus introducing an additional coherent loss mechanism in the system (the length of the fiber spans is assumed to be small so that the linear loss of the fiber itself can be neglected). The purpose of the SOA and AOM in the short and long arms of the second loop is to introduce the balanced gain *e*^*γ*^ and loss *e*^−*γ*^ respectively (*γ* ≥ 0), with a similar idea exploited in ref.^17^ in order to implement a parity-time symmetric discrete time quantum walk. Here however we will be using it to provide balanced hopping amplitudes of the Hatano-Nelson model^2^. The role of the two PMs is to implement a random clock model first discussed by Feinberg and Zee^26^. One can use a single PM in the main loop instead which will correspond to the “synchronized” clock model. Both active and phase elements can be removed/deactivated to realize either pure HN or RC case. The Methods section shows how the setup shown in Fig. 1 leads to the suggested model (1).

Note that if one uses an additional gain in the main loop as was assumed in ref.^15^ then in Eq. (1) there is an additional prefactor $\sqrt{g}$ which however can be put to unity by introducing the rescaled amplitude via: ${a}_{n}^{m}\to {g}^{m/2}{a}_{n}^{m}$ so it will be omitted in the following. It is also possible to track the evolution of the losses ${q}_{n}^{m}$ but this is beyond the scope of this paper. The presence of the positive gain *γ* in the shorter loop and a symmetric loss in the longer loop act as a bias shifting the pulse towards the decreasing values of of *n*. Needless to say that this only one possible setup for observing the effect discussed in this paper and from now on we concentrate only on theoretical analysis of the model leaving the experimental implementation to future studies.

### The spectral and localization properties of the evolution operator

In this section we study the properties of the step evolution operator defined by the r.h.s. of Eq. (2). We start from the known results for its special cases: the pure HN^2^ and RC^26,27^ models.

The solution of the pure HN model can be easily obtained^2^. For periodic BCs one has:

3 $${z}_{j}=\,\cosh \,\gamma \,\cos \,{k}_{j}+i\,\sinh \,\gamma \,\sin \,{k}_{j},\,{u}_{n}^{(j)}={N}^{-1/2}\,\exp (i{k}_{j}n)$$

with the effective Bloch wavevector *k*_*j*_ = 2*πj*/*N*, *j* = 0, …, *N* − 1. The eigenspectrum of the HN model is therefore complex with the eigenvalues populating the ellipse with the center at the origin and with semi-major (*x*) and semi-minor (*y*) axes given by cosh*γ* and sinh*γ* respectively. All the eigenmodes of the HN model are extended and clearly form a complete orthogonal set. In the Hermitian limit *γ* → 0 or in the case of free boundary conditions the ellipse collapses onto the real line Re[*z*] ∈ [−1, 1].

In the opposite limit when *γ* = 0 and Φ_*max*_ = *π* one obtains the “random clock” (RC) model^26,27^. The eigenspectrum of the random clock model is isotropic, approximately uniform and can be exactly shown to be contained inside the unit circle although the numerical simulations seem to indicate much tighter support radius of ≈*π*/4^26,27^. All the eigenmodes in this model (apart from a zero mode) are localized. It is possible in principle to implement other, more exotic or simpler models, e.g. “random sign model” also from ref.^26^ where the phases are restricted to the to values {0,*π*} or synchronized clocks model where *ϕ*_↑_(*n*) = *ϕ*_↓_(*n*).

What characterises both HN and RC models implemented as stroboscopic evolutionary operators is that neither of them can support stabilized pulse propagation for an arbitrary initial condition. For the RC model the spectral support is inside the unit circle and all the modes are decaying and it will require external amplification in the main loop to make at least some of the modes stable. Unless one engineers an initial condition overlapping only with these stable modes the total energy of the pulse will either decay or grow exponentially. On the other hand in the pure HN model the 1D elliptical spectral manifold will generally intersect the stationary region |*z*| = 1 only at four points, again meaning that only a limited amount of the modes will remain quasistationary and do not decay or grow exponentially.

**Figure 2:**
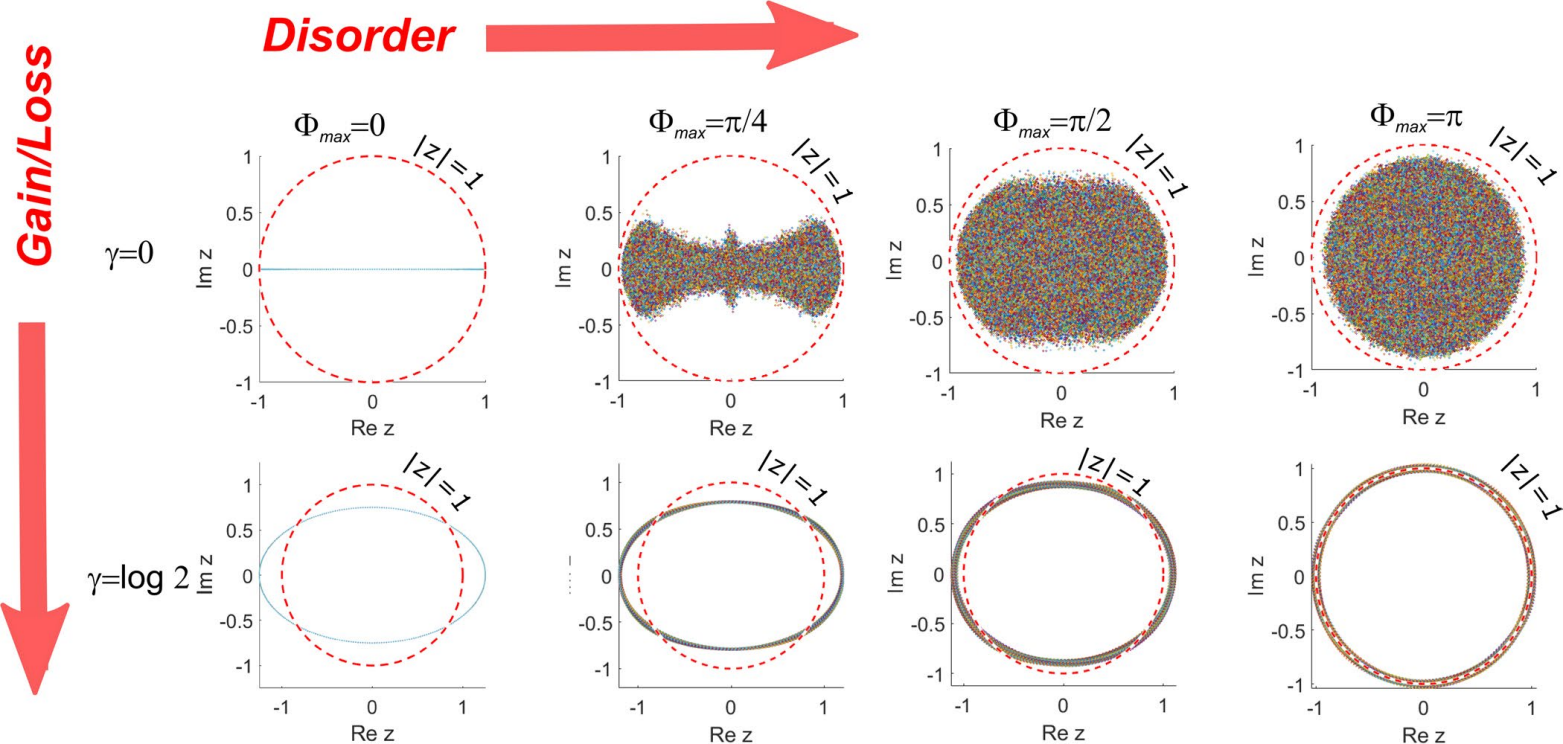
The support of eigenvalues of system (2) for the increasing values of disorder (left to right) for pure random clock model (top) and GHN (bottom). The stability margin |*z*| = 1 is shown by dashed red line.

We now turn to the spectral properties of the general GHN model. Apart from the system size *N* and PBC the model is completely characterized by the disorder strength 0 ≤ Φ_*max*_ < *π* and the gain/loss parameter *γ*. To study the spectrum of the GHN operator in more detail we have numerically diagonalized 1000 instances of 200 × 200 random matrices $\hat{U}$. Figure 2 shows the received support of the spectrum for four consecutive values of Φ_*max*_ (different columns). The top row corresponds to the pure RC model and the bottom one is a more general case of GHN with the of gain/loss increment *γ* = ln2. Pure HN case (Φ_*max*_ = 0) corresponds to the bottom left corner with the undistorted HN-ellipse. As discussed above in the absence of gain in the main loop the marginal stability region in the complex space of the complex spectral parameter corresponds to the unit circle: |*z*| = 1 (shown in Fig. 2 as a reference). For all the modes inside this region the power decays exponentially with the number of the round trips *m* as ~exp(2ln|*z*|*m*) and for the modes outside there is a similar exponential growth.

In the case of random clock model one can see that as the level of disorder progressively grows the spectrum invades the complex plane and gradually becomes more and more isotropic^26,27^. In fact it follows immediately from (2) that multiplying the spectral parameter *z* by a phase factor exp(*iϕ*) amounts to multiplying both “clocks” exp(*iϕ*_↑↓_(*n*)) by the same factor which in the case of full disorder Φ_*max*_ = *π* does not change their statistics so in this case the spectra are isotropic for both *γ* = 0 and *γ* > 0 case - see the last column in Fig. 2. What however makes the GHN model remarkable is that starting from the original HT ellipse in the case of low phase disorder the spectrum remains tightly localized while it continuously deforms into a circle as Φ_*max*_ approaches the maximum value of *π*. Such a behavior is a consequence of an extreme sensitivity of the GHN and its precursor HN model to the boundary conditions^2,26^. For example for the free (i.e. zero) boundary conditions *u*_0_ = *u*_*N*+1_ = 0 GHN reduces to the random clock model similarly as HN reduces to the Hermitian tight binding model (see the corresponding discussion in the Methods section).

Now let us look into the localization properties of the GHN eigenmodes. There are several basic tools for studying localization in both Hermitian and non-Hermitian systems but here we only use two of them. The first one is the so-called Lyapunov exponent (LE) widely used in Heremitian systems and also considered in the RC model in ref.^27^. The second one is recently suggested topological number test specifically applicable to the non-Hermitan systems^25^. We start with the LE. It can be defined by several equivalent ways either by considering the exponential spread of the norm of the 2 × 2 transfer matrix relating the two pairs of values *u*_0_, *u*_1_ and *u*_*N*−1_, *u*_*N*_ at the opposite boundaries of the SPL or via the limiting behavior of the so called so called Riccati variable defined as *r*_*n*_ = *u*_*n*_/*u*_*n*−1_^5,20,27^. Both iterative maps are determined by the Eq. (2) and definition of the LE is then

4 $$\lambda (z)=\frac{1}{N}\mathop{\sum }\limits_{n=1}^{N}\,\log \,|{r}_{n}|$$

where the limit *N* → ∞ is implied. Positive values of the LE inside the spectral region signify localized eigenmodes with the localization length given by the reciprocal value of *λ*(*z*), vanishing LE corresponds to delocalization. An important result here (see e.g.^5^) is the relation between the spectral and localization properties of the general biased models like (2) and the the unbiased ones with *γ* = 0. Namely, the explicit dependence of the LE on *γ*, *λ* = *λ*(*z*; *γ*) is simply:

5 $$\lambda (z;\gamma )=\lambda (z;0)-\gamma $$

This can be easily seen making a gauge transformation *u*_*n*_ = exp(−*γn*)*ψ*_*n*_ (see the Methods section). An important consequence of this result is that for the periodic boundary conditions the region where *λ*(*z*; *γ*) < 0 corresponds to non-normalizable eigenstates and hence this region corresponds to spectral hole well documented in literature (see e.g.^5,34^). This hole is not present in the free boundary case where the effects of gain loss/can be gauged out and the spectrum coincides with that of the pure RC model (see Fig. 2).

The LE of the RC was studied in ref.^27^ where it was shown that in the limit of large system size *N* the spectrum and localization properties are independent of the boundary conditions and all the states (apart from *z* = 0) are localized and *λ*(*z*; 0) ≥ 0 is growing monotonically with |*z*|. Thus the hole boundary is given by the condition *λ*(*z*; 0) = *γ* and near the rim of the hole *λ*(*z*; *γ*) ≈ 0 and all the states are delocalized while as |*z*| grows so does the *λ*(*z*; *γ*) according to the result (5). The boundary of this region can be estimated for the case of strong disorder Φ_*max*_ = *π*.

This can be done as follows. Since the spectrum of the unbiased random clock model vanishes outside the circle |*z*| = 1 it follows from the Thouless formula for non-Hermitian Jacobi matrices^27^ that in the region |*z*| > 1 the LE is given by *λ*(*z*; 0) = log(2|*z*|). Using this result for the first term in (5) one obtains the lower bound for the support of the spectrum:

6 $$|z{|}_{min}=r\ast =\frac{1}{2}{e}^{\gamma }\ge 1,\,\gamma  > \,\mathrm{ln}\,2$$

For small bias *γ* < log2 this criterion is inapplicable and one can only adduce that |*z*|_*min*_ must be a number between 0 and 1 since for all |*z*| > 1 it follows that *λ*(*z*; *γ*) > 0. On the other hand by making use of triangle inequality for |*u*_*n*_| = max_*n*_|*u*_*n*_| it follows from (2) that the upper bound for the spectral support is given by |*z*_*max*_| = cosh*γ*.

In the limit of large *γ* both upper and lower bounds coalesce i.e. all the eigenvalues are located on the same circle of the radius r_*_ given by Eq. (6). To study the localization properties of the eigenmodes in this case consider system (2) but neglecting the reverse bias terms exp(−*γ*). Solving the system recursively one obtains the solution as ${u}_{n}={(2z{e}^{-\gamma })}^{n-1}\exp (-i\mathop{\sum }\limits_{k=1}^{n-1}\,{\varphi }_{\uparrow }(k)){u}_{1}$ where *u*_1_ is determined by normalization. Using PBC one obtains the spectrum as:

7 $${z}_{j}=r\ast {({e}^{i\mathop{\sum }\limits_{k=1}^{N}{\varphi }_{\uparrow }(k)})}^{1/N}=r\ast {e}^{i\Delta \varphi +i{k}_{j}}$$

with the $\Delta \varphi ={N}^{-1}\mathop{\sum }\limits_{n=1}^{N}\,{\varphi }_{\uparrow }(n)$ being the spatially averaged phase distribution and *k*_*j*_ is the same Bloch vector as in (3). One can see that regardless of the phase modulation all the eigenvalues given by (7) are located on the same circle of radius r_*_ while the eigenstates are extended. Disorder only makes the angular distance between the eigenvalues random but otherwise has no effect on the spectral and localization properties. Higher order corrections can also be extracted following the procedure similar to that outlined in^5^.

Thus at large gain/loss all the modes are extended and the system is in the lasing regime with the amplitude of all the modes growing at the same rate lnr_*_ = *γ* − ln(2) (the latter term is due to the 50% power loss at the second coupler - see Fig. 1). However when *γ* = ln2 the system is in a quaistationary regime where the gain in the shorter arm compensates the detection loss at the second coupler. This compensation is not exact since while the bound (6) remains tight the approximation (7) (corresponding to neglecting the contribution of the longer loop) is not strictly applicable. In the pure HN it is the contribution of this second (damped) loop in-phase with that of the amplified loop that makes the pure HN spectrum asymmetric and hence only a fraction of modes can be stabilized at moderate values of the gain. The presence of disorder however changes the picture dramatically since the there is no longer phase coherence between the advanced and retarded pulses and hence the contribution of the damped loop into the pulse dynamics is further reduced thus extending the applicability of the result (7) towards smaller gain.

Let us now move to the topological criteria for the presence or absence of Anderson transition in system (2). It was recently conjectured in^25^ (see also^33^) that the total localization or delocalization of all modes in the non-Heremitian systems of the HN type can be indicated by considering a topological charge (a winding number) defined as^25,33^:

8 $$w=\frac{1}{2\pi \,i}{\int }_{0}^{2\pi }\,d\theta \,\frac{\partial }{\partial \theta }\,\mathrm{ln}\,{\rm{\det }}\,U(\theta ),$$

where *U*(*θ*) is a gauge-transformed Floquet operator obtained from *U* by replacing the gain *γ* with *γ* + *iθ*/*N*, where *N* is the period of the system. It was argued that as the effective “magnetic flux” *θ* changes between 0 and 2*π* the eigenvalues corresponding to the localized modes remain spectrally rigid. Therefore in a system where all the modes are localized the spectrum flow defining the winding number is trivially zero. However in the presence of however small amount of delocalized modes their eigenvalue move with *θ* and the winding number changes to ±1 (depending on the definition of the sign of the phase) signifying delocalization transition. Using this simple argument and the calculation of the winding number in^25^ and^33^ the authors obtained a simple verification of the Anderson transition occurring at pure HN model at the critical strength of the diagonal disorder and/or gain.

**Figure 3:**
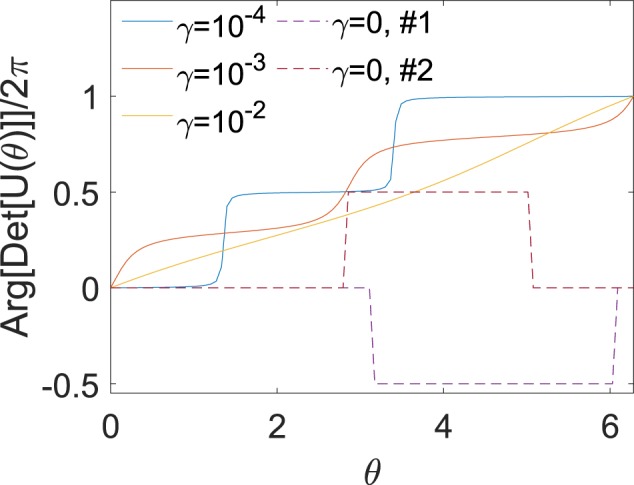
The argument flow, Arg (det*U*(*θ*)) in case of strong phase disorder Φ_*max*_ = *π* for the GHN (*γ* > 0, solid lines) and RC (*γ* = 0, dashed lines) models. Five representative realizations are shown.

However the arguments of ref.^25^ addressed the model where the complex nearest coupling terms of the Hamiltonian maintained translational invariance and disorder was purely diagonal. It is interesting therefore to look at the topological picture of system (2) with an off-diagonal phase disorder that apparently belongs to entirely different class. We therefore performed multiple simulations where for each value of the gain parameter *γ* and for each realization of phase disorder we have numerically diagonalized matrices of 200 sites on a grid of “effective fluxes” *θ* and considered the dependence of Arg (det[*U*(*θ*)]) (also called the argument flow in^25^) after subtracting the initial value at zero flux. It is the total increment of this quantity (modulo 2*π*) that defines the winding number. The results were that we could not observe a topological transition for a finite value of the gain/loss *γ* and even for the maximal level of phase disorder (Φ_*max*_ = *π*). In all the simulations and all realization of the phase the winding number was always detected to be 1 in a lossy system (*γ* > 0) - signalling the presence of a finite amount of delocalized states. This is in stark contrast with the system with diagonal disorder where the topological transition does occur^25,33^. In Fig. 3 we present five trajectories of the argument flow corresponding to five different realizations of phase disorder and different values of the gain parameter *γ* raging from zero to 10^−2^. Larger values of *γ* yield almost constant positive slope 1/2*π* yielding the total accumulated topological charge *w* = 1.

These results are of course in full agreement with the Lyapunov analysis above, since we have seen that the states near the rim of the spectral hole are always delocalized as their LE vanishes. Since the hole exists for arbitrary small values of the gain parameter *γ* one can indeed see that the complete Anderson transition is impossible in these systems for *γ* > 0. This is also now confirmed by a topological argument. For the reference we also provide a graph for a couple of realizations of a pure random clock model *γ* = 0 where it is well known that the LE vanishes at the origin *z* = 0^27^ - i.e. the rim of the ring collapses into a single origin point with the zero eigenmode being delocalized. In fact in a periodic system one can always explicitly construct this zero mode for arbitrary distribution of random hopping elements (provided that the gain is zero) and show that it is extended^5^. One can observe from Fig. 3 that the argument flow of the RC model jumps between zero and ±*π* at random positions always returning to the original level at *θ* = 2*π* so that the total increment (and hence the winding number) is zero which might suggest fully localized regime. This is of course a wrong conclusion since the results of^25^ are strictly applicable for the spectrum bounded away from a fixed point (zero in this case) - a condition violated for the RC model. Indeed one can see from Eq. (8) that for degenerate matrices where one or more of the eigenvalues vanish at arbitrary flux *θ* the argument of the log is ill-defined which causes random jumps in the argument flow.

### Dynamical pulse stabilization and steering

**Figure 4:**
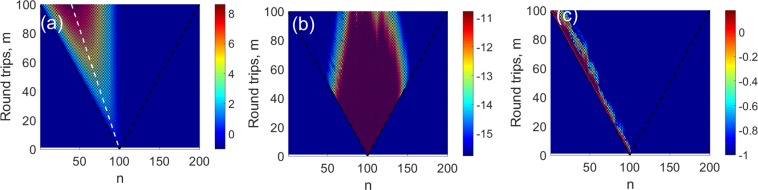
Propagation of an initially localized pulse in different models (log_10_|*a*_*n*_| shown): (**a**) Pure HN model (Φ_*max*_ = 0, *γ* = ln2), (**b**) RC (Φ_*max*_ = *π*, *γ* = 0), (**c**) GHN with ring topology (Φ_*max*_ = *π*, *γ* = ln2). The black dashed lines bound a causality cone while the white line in (**a**) marks the theoretical prediction of the group velocity (see main text).

Finally let us now study the dynamical properties in all of the three cases described by the evolutionary dynamics (1). These include pure HN case (Φ_*max*_ = 0), random clock model (*γ* = 0) and the optimal stabilization regime *γ* = ln2. As we shall see our main result is the demonstration of the effect of pulse stabilization in the case of strong disorder, Φ_*max*_ = *π* with periodic boundary conditions and optimal gain coefficient *γ* = ln2 as was predicted from the spectral properties of the Floquet operator discussed above. To this end we have simulated the propagation of the pulse ${a}_{n}^{m}$ from Eq. (1) starting from a point excitation ${a}_{n}^{0}={\delta }_{n{n}_{0}}$. In all cases the system size was *N* = 200. The results are shown in Fig. 4. Two causal lines with the slope ±1 are shown for the reference.

One fact that is immediately noticeable in Fig. 4(c) is that disorder leads not only to pulse stabilization but also to strong steering with the group velocity close to the casual one even for moderate bias *γ* = ln2 - again something that is not observed in the deterministic model - c.f. Fig. 4(a). To understand this let us derive the expression for the group velocity of the dispersing wave packet for pure HN model. Given that the modes ${u}_{n}^{(j)}$ of pure HN model (3) form a complete orthogonal set the solution of (1) with the initial point excitation is given by:

9 $${a}_{n}^{m}=\mathop{\sum }\limits_{j=0}^{N-1}\,\,{(i{z}_{j})}^{m}\,{u}_{{n}_{0}}^{\ast (j)}\,{u}_{n}^{(j)}=\mathop{\sum }\limits_{j=0}^{N-1}\,\frac{{(i{z}_{j})}^{m}}{N}\,{e}^{i{k}_{j}(n-{n}_{0})}.$$

At large *m* the main contributions comes from the amplified modes near the longer tips of the HN-ellipse corresponding to the points *k*_*j*_ = *πn*, *n* = 0, 1. To obtain the group velocity of the driving we need to expand the eigenvalues *z*(*k*_*j*_) = *z*_*j*_ near these points. One has up to the first nonvanishing order in the amplitude and phase

$$z(\pi n+\Delta k)\approx {(-1)}^{n}\,\cosh (\gamma )\,{e}^{-{(\Delta k)}^{2}/(2{\cosh }^{2}\gamma )}\,{e}^{i\Delta k\tanh \gamma }$$

The solution ${a}_{n}^{m}$ at large *m* therefore represents a narrow dispersing wavepacket with a spectral width Δk_*_(*m*) = cosh^2^*γ*/*m* moving with a group velocity *v*_*g*_ = −tanh*γ* - shown in Fig. 4(a) as a white dashed line.

Now let us move to the disordered case. For *γ* ≫ 1 one can approximate the solution of the spectral problem by that obtained in the previous section neglecting the reverse bias the solution of which is given by (7). The spectrum is equidistant so that Arg(*z*_*j*_) = Δ*ϕ* + *k*_*j*_. The modes are given by ${u}_{n}^{(j)}=({C}_{n}/\sqrt{N})\,\exp (i{k}_{j}(n-1))$ where the coefficients *C*_*n*_ are random phase factors that are the same for each mode. It is important that similarly to the pure HN case these eigenmodes also form a complete orthogonal set so that one can again use a modal expansion (9) and obtain

$${a}_{n}^{m}={i}^{m}{r}_{\ast }^{m}{e}^{i\Delta \varphi m}{C}_{{n}_{0}}^{\ast }{C}_{n}\sum _{j}\,{N}^{-1}{e}^{i{k}_{j}(n-{n}_{0}+m)}={i}^{m}{r}_{\ast }^{m}{e}^{i\Delta \varphi m}{C}_{{n}_{0}}^{\ast }{C}_{n}\,{\delta }_{n,{n}_{0}+m}$$

In the limit of balanced gain, *γ* = ln2, r_*_ = 1 and thus up to irrelevant phase factor the solution maintains its localized shape and moves with the effective group velocity equal to −1 -the maximum possible one - with a good agreement with the simulations (see Fig. 4c). Note that this happens despite most of the eigenmodes being delocalized in this limit. In reality for *γ* = ln2 one can observe both small spread and deviation of group velocity for −1 which is a natural consequence of the approximate nature of Eq. (7) which is valid only for exp(*γ*) ≫ 1. In the latter regime however we expect the above result to hold but the total power is now growing as exp(2*m*ln|r_*_|) = exp(2*m*(*γ* − ln2)).

**Figure 5:**
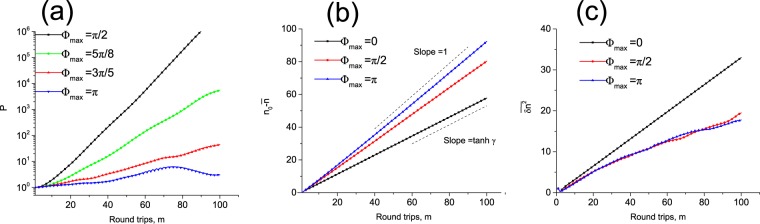
Evolution of the first three moments of field distribution for the progressively increasing values of phase disorder.

To quantify both steering and spreading of the localized excitation we have performed additional numerical simulations where we have studied temporal evolution of the following quantities:

10 $$P(m)=\sum _{n}\,|{a}_{n}^{m}{|}^{2},\,\bar{n}(m)=\frac{{\sum }_{n}\,n|{a}_{n}^{m}{|}^{2}}{P(m)},\,\overline{\delta {n}^{2}}(m)=\frac{{\sum }_{n}\,(n-\bar{n}(m))|{a}_{n}^{m}{|}^{2}}{P(m)}$$

for different values of disorder. In each run we have chosen the value of gain slightly sub-threshold *γ* = ln2 − 0.03 in order to prevent some of the modes from crossing into the unstable region |*z*| > 1. The first quantity is just the total power (norm) of the solution while the the other two represent spatial average and variance of the wavepacket: $\bar{n}(m)$ is the position of the centre of mass and $\overline{\delta {n}^{2}(m)}$ is a measure of transverse spread (dynamical localization). The results (averaged over 500 realizations of disorder) are shown in Fig. 5.

As seen in Fig. 5(a) small values of disorder cannot stabilize the pulse due to the proliferation of the unstable modes (see the bottom row of Fig. 2). However as the level of disorder is increased the exponential growth of the norm *P*(*m*) is virtually arrested. In Fig. 5(b) in all cases one can see an almost uniform motion of the centre of mass of the wave-packet (plotted here relative to the initial position) with the slope progressively increasing from tanh*γ* to 1 with the growth of disorder as predicted by theory. Finally in Fig. 5(c) the spread of pulse is studied. For disorder free propagation the spread of the wave-packet is diffusive $\overline{\delta {n}^{2}}\propto m$ as follows from Eq. (9) and the growth slows down as disorder grows. However as discussed above, the majority of the eigenmodes in the system are extended and no dynamical localization is expected (at least for a finite system with ring topology).

## Discussion

We have studied the interplay of disorder and gain/loss in a synthetic photonic lattices realizing stroboscopic evolution of several known non-Hermitian/non-unitary models. We have shown that high levels of disorder can actually stabilize pulse propagation and make the pulse evolution quasi-unitary despite the system lacking any corresponding symmetry (for example PT). This stabilization is accompanied by pulse steering with the maximum group velocity independent of the gain/loss.

## Methods

### Derivation of the model (1) for the suggested setup

Let us represent the amplitude of signal in the main loop after *m* round trips at the position *n* as ${a}_{n}^{m}$. The pulse is initially injected at a time mark *n*_0_ and upon entering the first coupler splits into the upper and lower arm each of which is shorter/longer than the main loop by a distance Δ*L* corresponding to a shift of of one temporal position *n* ± 1. Because of that after one round trip the pulse from the position *n* in the main loop entering the shorter arm overshoots arriving at the position *n* − 1 and the pulse entering the longer loop undershoots arriving at *n* + 1. Introducing the auxiliary amplitudes ${u}_{n}^{m}$ and ${v}_{n}^{m}$ for the upper and lower arms respectively and taking into account the active and phase elements and the fact that that the second port of the first coupler is fed with vacuum one obtains ${u}_{n}^{m}=i\,\exp (i{\varphi }_{\uparrow }(n))\,\exp (\gamma )\,{a}_{n+1}^{m}/\sqrt{2}$, ${v}_{n}^{m}=\exp (i{\varphi }_{\downarrow }(n))\,\exp (\,-\,\gamma )\,{a}_{n-1}^{m}/\sqrt{2}$. Here *ϕ*_↑↓_(*n*) are the static (i.e. constant between the round trips) phases imposed by phase modulator in the shorter/longer arms of the inner loop (see e.g.^15,19^). The two output ports of the second coupler correspond to the amplitude dissipated at the detector ${q}_{n}^{m+1}$ and the main loop amplitude after single discrete time step ${a}_{n}^{m+1}$. Thus we obtain the final evolution model (1) for the signal.

### Spectrum sensitivity for boundary conditions

Unlike passive models corresponding to Hermitian effective hamiltonian ${H}_{eff}^{\dagger }={H}_{eff}$ the spectra and localization properties of GHN and its precursor HN model are extremely sensitive to the boundary conditions^2,26^. In order to see this we introduce a standard “imaginary gauge” transformation *u*_*n*_ = exp(−*γn*)*ψ*_*n*_ and for the new field and PBC one obtains the eigenvalue for the transformed matrix $\hat{U}^{\prime}$:

11 $$z{\psi }_{n}=\frac{1}{2}\,[{e}^{i{\varphi }_{\uparrow }(n)}\,{\psi }_{n+1}+{e}^{i{\varphi }_{\downarrow }(n)}\,{\psi }_{n-1}],\,{\psi }_{0}={e}^{-\gamma N}\,{\psi }_{N},\,{\psi }_{N+1}={e}^{\gamma N}\,{\psi }_{1}$$

Clearly model (11) is isospectral with (2) and moreover the main body of the new matrix $\hat{U}^{\prime}$ is identical to that of the passive RC model save for the exponentially large/small “corners” introduced by the PBC^5,34^. For the passive RC model with PBC these corners exist also but they are *O*(1) so the choice of the boundary conditions does not affect the spectrum in the thermodynamic limit *N* → ∞^27^. For the non-zero *γ* however one must distinguish between the free boundary conditions (where the matrix is purely tridiagonal and the spectrum is identical to the pure RC, top right corner of Fig. 2) and PBC that lead to the spectral confinement and pulse stabilization. For the pure RC model the spectrum is insensitive to the BC with the eigenvalues are uniformly spread inside a circle with each mode decaying as *ψ* ~ exp(−*λ*(*z*, 0)|*n* − *n*_0_|). Therefore the eigenmodes of the GHN with the free boundary conditions are decaying as *u*_*n*_ ~ exp(−*γn* − *λ*(*z*, 0)|*n* − *n*_0_|) where *λ*(*z*, 0) is the Lyapunov exponent of the RC model. For example the eigenmodes near the origin *z* = 0 have vanishingly small LE and that their localization length is determined by *γ*^−1^. In the PBC case there is no longer a connection between the shape of the eigenmodes of the GHN and that of the RC model and, as discussed in the main text a hole opens in the spectrum and almost all the eigenmodes are extended.

## Data Availability

The datasets generated during and/or analysed during the current study are available from the author on reasonable request.
